# Traditional plant functional groups explain variation in economic but not size‐related traits across the tundra biome

**DOI:** 10.1111/geb.12783

**Published:** 2018-11-16

**Authors:** H. J. D. Thomas, I. H. Myers‐Smith, A. D. Bjorkman, S. C. Elmendorf, D. Blok, J. H. C. Cornelissen, B. C. Forbes, R. D. Hollister, S. Normand, J. S. Prevéy, C. Rixen, G. Schaepman‐Strub, M. Wilmking, S. Wipf, W. K. Cornwell, J. Kattge, S. J. Goetz, K. C. Guay, J. M. Alatalo, A. Anadon‐Rosell, S. Angers‐Blondin, L. T. Berner, R. G. Björk, A. Buchwal, A. Buras, M. Carbognani, K. Christie, L. Siegwart Collier, E. J. Cooper, A. Eskelinen, E. R. Frei, O. Grau, P. Grogan, M. Hallinger, M. M. P. D. Heijmans, L. Hermanutz, J. M. G. Hudson, K. Hülber, M. Iturrate‐Garcia, C. M. Iversen, F. Jaroszynska, J. F. Johnstone, E. Kaarlejärvi, A. Kulonen, L. J. Lamarque, E. Lévesque, C. J. Little, A. Michelsen, A. Milbau, J. Nabe‐Nielsen, S. S. Nielsen, J. M. Ninot, S. F. Oberbauer, J. Olofsson, V. G. Onipchenko, A. Petraglia, S. B. Rumpf, P. R. Semenchuk, N. A. Soudzilovskaia, M. J. Spasojevic, J. D. M. Speed, K. D. Tape, M. te Beest, M. Tomaselli, A. Trant, U. A. Treier, S. Venn, T. Vowles, S. Weijers, T. Zamin, O. K. Atkin, M. Bahn, B. Blonder, G. Campetella, B. E. L. Cerabolini, F. S. Chapin III, M. Dainese, F. T. de Vries, S. Díaz, W. Green, R. B. Jackson, P. Manning, Ü. Niinemets, W. A. Ozinga, J. Peñuelas, P. B. Reich, B. Schamp, S. Sheremetev, P. M. van Bodegom

**Affiliations:** ^1^ School of Geosciences University of Edinburgh Edinburgh United Kingdom; ^2^ Ecoinformatics and Biodiversity, Department of Bioscience, Aarhus University Aarhus Denmark; ^3^ Senckenberg Gesellschaft für Naturforschung, Biodiversity and Climate Research Centre (SBiK‐F) Frankfurt Germany; ^4^ Institute of Arctic and Alpine Research, University of Colorado Boulder Colorado; ^5^ Department of Physical Geography and Ecosystem Science, Lund University Lund Sweden; ^6^ Department of Ecological Science, Vrije Universiteit Amsterdam The Netherlands; ^7^ Arctic Centre, University of Lapland Rovaniemi Finland; ^8^ Biology Department, Grand Valley State University Allendale Michigan; ^9^ WSL Institute for Snow and Avalanche Research SLF Davos Switzerland; ^10^ Department of Evolutionary Biology and Environmental Studies, University of Zurich Zurich Switzerland; ^11^ Institute for Botany and Landscape Ecology, Greifswald University Greifswald Germany; ^12^ School of Biological Earth and Environmental Sciences, University of New South Wales Sydney New South Wales Australia; ^13^ Max Planck Institute for Biogeochemistry Jena Germany; ^14^ German Centre for Integrative Biodiversity Research (iDiv) Halle‐Jena‐Leipzig Germany; ^15^ School of Informatics, Computing, and Cyber Systems, Northern Arizona University Flagstaff Arizona; ^16^ Bigelow Laboratory for Ocean Sciences Boothbay Maine; ^17^ Department of Biological and Environmental Sciences, Qatar University Doha Qatar; ^18^ Department of Evolutionary Biology, Ecology and Environmental Sciences, University of Barcelona Barcelona Spain; ^19^ Biodiversity Research Institute University of Barcelona Barcelona Spain; ^20^ Department of Earth Sciences, University of Gothenburg Gothenburg Sweden; ^21^ Gothenburg Global Biodiversity Centre Gothenburg Sweden; ^22^ Institute of Geoecology and Geoinformation, Adam Mickiewicz University Poznan Poland; ^23^ Department of Biological Sciences, University of Alaska Anchorage Anchorage Alaska; ^24^ Forest Ecology and Forest Management, Wageningen University and Research, Wageningen Netherlands; ^25^ Department of Chemistry, Life Sciences and Environmental Sustainability, University of Parma Parma Italy; ^26^ The Alaska Department of Fish and Game Juneau Alaska; ^27^ Department of Biology, Memorial University St John’s, Newfoundland and Labrador Canada; ^28^ Department of Arctic and Marine Biology, UiT‐The Arctic University of Norway Tromsø Norway; ^29^ Department of Physiological Diversity, Helmholtz Centre for Environmental Research – UFZ Leipzig Germany; ^30^ Department of Ecology and Genetics, University of Oulu Oulu Finland; ^31^ Department of Geography, University of British Columbia Vancouver British Columbia Canada; ^32^ Global Ecology Unit, CREAF‐CSIC‐UAB‐UB Bellaterra Spain; ^33^ Department of Biology, Queen's University Kingston, Ontario Canada; ^34^ Biology Department, Swedish Agricultural University (SLU) Uppsala Sweden; ^35^ Plant Ecology and Nature Conservation Group, Wageningen University & Research Wageningen The Netherlands; ^36^ British Columbia Public Service British Columbia Canada; ^37^ Department of Botany and Biodiversity Research, University of Vienna Vienna Austria; ^38^ Climate Change Science Institute and Environmental Sciences Division, Oak Ridge National Laboratory Oak Ridge Tennessee; ^39^ Department of Biology, University of Bergen Bergen Norway; ^40^ Department of Biology, University of Saskatchewan Saskatoon Canada; ^41^ Department of Ecology and Environmental Sciences, Umeå University Umeå Sweden; ^42^ Department of Biology, Vrije Universiteit Brussel (VUB) Brussels Belgium; ^43^ Faculty of Biological and Environmental Sciences, University of Helsinki Helsinki Finland; ^44^ Département des Sciences de l'Environnement and Centres d'études nordiques, Université du Québec à Trois‐Rivières Trois‐Rivières Quebec Canada; ^45^ Eawag Swiss Federal Institute of Aquatic Science & Technology Dubendorf Switzerland; ^46^ Department of Biology, University of Copenhagen Copenhagen Denmark; ^47^ Center for Permafrost (CENPERM), University of Copenhagen Copenhagen Denmark; ^48^ Research Institute for Nature and Forest (INBO) Brussels Belgium; ^49^ Department of Biological Sciences, Florida International University Miami Florida; ^50^ Department of Geobotany, Lomonosov Moscow State University Moscow Russia; ^51^ Environmental Biology, Department Institute of Environmental Sciences, CML, Leiden University Leiden The Netherlands; ^52^ Department of Biology, University of California Riverside Riverside California; ^53^ NTNU University Museum, Norwegian University of Science and Technology Trondheim Norway; ^54^ Water and Environmental Research Center, University of Alaska Fairbanks Alaska; ^55^ Environmental Sciences, Copernicus Institute of Sustainable Development, Utrecht University Utrecht The Netherlands; ^56^ School of Environment, Resources and Sustainability, University of Waterloo Waterloo Ontario Canada; ^57^ Research School of Biology, Australian National University Acton, ACT Australia; ^58^ Centre for Integrative Ecology, School of Life and Environmental Sciences, Deakin University Burwood Victoria Australia; ^59^ Department of Geography, University of Bonn Bonn Germany; ^60^ Department of Ecology, University of Innsbruck Innsbruck Austria; ^61^ Environmental Change Institute, School of Geography and the Environment, University of Oxford Oxford United Kingdom; ^62^ Rocky Mountain Biological Laboratory Crested Butte Colorado; ^63^ School of Biosciences & Veterinary Medicine ‐ Plant Diversity and Ecosystems Management Unit, University of Camerino Camerino Italy; ^64^ DiSTA, University of Insubria Varese Italy; ^65^ Institute of Arctic Biology, University of Alaska Fairbanks Alaska; ^66^ Department of Animal Ecology and Tropical Biology, University of Würzburg Würzburg Germany; ^67^ School of Earth and Environmental Sciences, The University of Manchester Manchester United Kingdom; ^68^ Instituto Multidisciplinario de Biología Vegetal (IMBIV), CONICET and FCEFyN, Universidad Nacional de Córdoba Córdoba Argentina; ^69^ Department of Organismic and Evolutionary Biology, Harvard University Cambridge, Massachusetts; ^70^ Department of Earth System Science, Stanford University Stanford, California; ^71^ Institute of Agricultural and Environmental Sciences, Estonian University of Life Sciences Tartu Estonia; ^72^ CREAF Cerdanyola del Vallès Spain; ^73^ Department of Forest Resources, University of Minnesota St. Paul, Minneapolis Minnesota; ^74^ Hawkesbury Institute for the Environment, Western Sydney University Penrith, NSW Australia; ^75^ Department of Biology, Algoma University Sault Ste. Marie Ontario Canada; ^76^ Komarov Botanical Institute St Petersburg Russia

**Keywords:** cluster analysis, community composition, ecosystem function, plant functional groups, plant functional types, plant traits, tundra biome, vegetation change

## Abstract

**Aim:**

Plant functional groups are widely used in community ecology and earth system modelling to describe trait variation within and across plant communities. However, this approach rests on the assumption that functional groups explain a large proportion of trait variation among species. We test whether four commonly used plant functional groups represent variation in six ecologically important plant traits.

**Location:**

Tundra biome.

**Time period:**

Data collected between 1964 and 2016.

**Major taxa studied:**

295 tundra vascular plant species.

**Methods:**

We compiled a database of six plant traits (plant height, leaf area, specific leaf area, leaf dry matter content, leaf nitrogen, seed mass) for tundra species. We examined the variation in species‐level trait expression explained by four traditional functional groups (evergreen shrubs, deciduous shrubs, graminoids, forbs), and whether variation explained was dependent upon the traits included in analysis. We further compared the explanatory power and species composition of functional groups to alternative classifications generated using post hoc clustering of species‐level traits.

**Results:**

Traditional functional groups explained significant differences in trait expression, particularly amongst traits associated with resource economics, which were consistent across sites and at the biome scale. However, functional groups explained 19% of overall trait variation and poorly represented differences in traits associated with plant size. Post hoc classification of species did not correspond well with traditional functional groups, and explained twice as much variation in species‐level trait expression.

**Main conclusions:**

Traditional functional groups only coarsely represent variation in well‐measured traits within tundra plant communities, and better explain resource economic traits than size‐related traits. We recommend caution when using functional group approaches to predict tundra vegetation change, or ecosystem functions relating to plant size, such as albedo or carbon storage. We argue that alternative classifications or direct use of specific plant traits could provide new insights for ecological prediction and modelling.

## 1 INTRODUCTION

Many ecosystems around the world are responding rapidly to global change drivers, including warming (IPCC, 2013), changing precipitation patterns (Weltzin et al., 2003), increased nutrient availability (Galloway et al., 2008), elevated atmospheric CO_2_ (Cramer et al., 2001) and altered herbivory regimes (Díaz et al., 2007). Perhaps nowhere will ecosystem response to climate change be greater than in the tundra, which is warming at twice the global average rate (IPCC, 2013; Serreze & Barry, 2011) and undergoing rapid vegetation change (Elmendorf, Henry, Hollister, Björk, Boulanger‐Lapointe, et al., 2012; Myers‐Smith et al., 2011). Predicting how plant communities will respond to environmental change, and the resulting impact on ecosystem structure and function, has been described as the “holy grail” of ecology (Lavorel & Garnier, 2002). However, the responses of different species and environments are often highly complex, representing a major challenge for the prediction of community response to environment change (Díaz et al., 2016; McGill, Enquist, Weiher, & Westoby, 2006).

**Figure 1:**
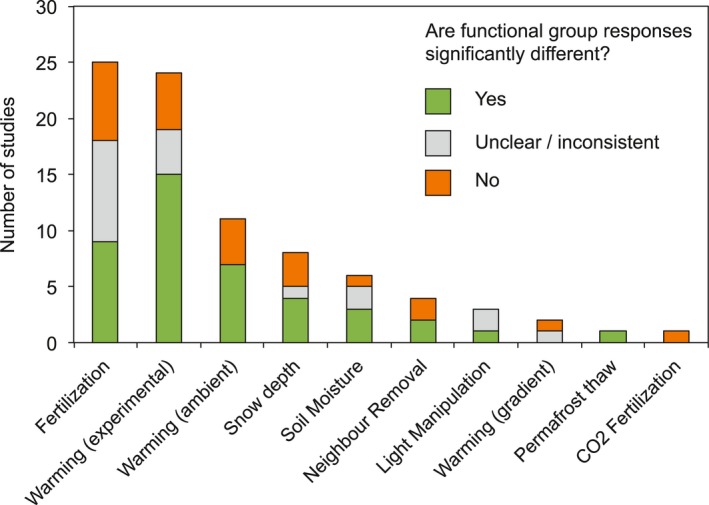
Studies employing an “evergreen shrub ‐ deciduous shrub ‐ graminoid – forb” functional group classification (or close variant) to examine the response of tundra communities to environmental change over the past two decades. Studies were identified based on a literature search on *Web of Science* using the search terms “tundra" and “plant functional group” or “plant functional type”. For a list of studies see Appendix A. Studies are grouped by whether they found clear differences in functional group response (*Yes*: clear differences were found between some (but not necessarily all) functional groups; *Not clear*: differences between groups were inconsistent amongst sites or over time; *No*: No significant differences in functional group response). Studies vary in duration from 2–30 years and incorporate a range of bioclimatic contexts and experimental types. For full meta‐analyses of functional group response see Dormann and Woodin (2002) and Dorrepaal (2007)

One approach to reducing complexity in ecological communities is to classify species with similar characteristics into plant functional groups or plant functional types (Harrison et al., 2010). Species are commonly grouped based on *a priori* classification by growth form (e.g., forb, shrub), life history (e.g., evergreen, deciduous) or other morphological characteristics (Wright et al., 2006; Wullschleger et al., 2014). In the tundra, vascular plant species are most commonly categorized into four functional groups: evergreen shrubs, deciduous shrubs, graminoids and forbs. This grouping structure is rooted in Chapin, Bret‐Harte, Hobbie, and Zhong’s (1996) demonstration that clustering of 37 species based on 21 plant traits aligned with growth form‐based groupings. The use of functional groups is thus inherently a trait‐based approach, based on the hypothesis that plant species within functional groups possess similar traits and act in ecologically similar ways (Lavorel & Garnier, 2002; McGill et al., 2006). This hypothesis has so far only been tested at the site scale (Chapin et al., 1996) or for individual traits (Dorrepaal, Cornelissen, Aerts, Wallén, & Logtestijn, 2005; Körner, Leuzinger, Riedl, Siegwolf, & Streule, 2016), yet continues to underpin a wide range of studies examining tundra plant community responses to environmental change (Figure 1).

There is evidence that functional groups display distinct differences in their response to environmental change in the tundra. Experimental warming and fertilization are associated with increases in cover and biomass of deciduous shrubs and graminoids, often at the expense of other functional groups (Dormann & Woodin, 2002; Elmendorf, Henry, Hollister, Björk, Bjorkman, et al., 2012). In turn, the relative abundance of different functional groups influences multiple ecosystem properties, including biomass accumulation, light interception, soil moisture and soil nutrients (McLaren & Turkington, 2010, 2011). Functional groups also integrate multiple plant traits and may therefore better explain ecosystem function and community change compared to single trait‐based approaches (Laughlin & Messier, 2015; Soudzilovskaia et al., 2013). By extension, plant functional groups may integrate information from traits that are difficult to collect, including root structure or mycorrhizal association, that may be critical to explaining vegetation change (Cornelissen, Aerts, Cerabolini, Werger, & Heijden, 2001; Soudzilovskaia et al., 2015).

Despite their prevalence in ecological analysis, functional groups have often displayed low explanatory power and inconsistent responses across experiments (Bret‐Harte et al., 2008; Dorrepaal, 2007). In a meta‐analysis of 36 environmental manipulation experiments in the tundra, Dormann and Woodin (2002) found that plant functional groups did not predict community response, except in the case of fertilization and warming treatments. Even amongst these treatment types, differences in functional group response have not always been clear in the literature (Figure 1). Functional groups have also shown highly conflicting responses across studies; for example, evergreen shrubs have shown positive, neutral and negative responses to warming (Elmendorf, Henry, Hollister, Björk, Boulanger‐Lapointe, et al., 2012; Hollister, Webber, & Tweedie, 2005; Zamin, Bret‐Harte, & Grogan, 2014). Finally, functional groups have shown inconsistent responses among and within experiments, in different years (Cornelissen & Makoto, 2014), time‐scales (Saccone & Virtanen, 2016), environmental conditions (Dorrepaal, 2007) and spatial scales (Mörsdorf et al., 2015).

Low explanatory power may arise from high trait variation within functional groups, such that group differences are not significant, particularly among small species pools (Cornelissen et al., 2004). For example, Körner et al. (2016) found that tissue carbon and nitrogen did not vary by functional group in European alpine plants, whilst Iversen et al. (2017) reported greater variation in fine‐root carbon‐to‐nitrogen ratios within groups than among groups in biomes spanning the globe. Many studies have instead found that tundra species respond highly individualistically to change (Hollister et al., 2005; Hudson, Henry, & Cornwell, 2011; Lavorel & Garnier, 2002), and that functional group responses instead reflect strong species‐specific responses, often of dominant species (Bret‐Harte et al., 2008; Little, Jagerbrand, Molau, & Alatalo, 2015; Shaver et al., 2001). An alternative hypothesis is, therefore, that traditional functional groups do not represent key dimensions of trait variation among species, and thus may obscure certain aspects of ecosystem function and change. Given that much of our current understanding of tundra vegetation change is based on functional group responses (Elmendorf, Henry, Hollister, Björk, Boulanger‐Lapointe, et al., 2012; McLaren & Turkington, 2010; Myers‐Smith et al., 2011), testing this hypothesis is critical to understanding the mechanisms and future patterns of tundra vegetation change.

### 1.1 Research questions

#### 1.1.1 How well do functional groups represent species trait variation?

In this study, we test whether traditional functional groups explain differences in six plant functional traits among Arctic and alpine tundra species, and whether explanatory power is sensitive to: (a) differences in species composition among sites or (b) the use of different plant traits in analyses. We examine six traits, plant height (PH), seed mass (SM), leaf area (LA), specific leaf area (SLA), leaf dry matter content (LDMC) and leaf nitrogen (LN), that are the most commonly collected plant traits in the tundra biome (Bjorkman et al., 2018a) and considered to be cornerstones of plant ecological strategy (Díaz et al., 2016). We hypothesize that plant functional groups will exhibit distinct trait distributions, and that traits associated with plant economics (SLA, LDMC, LN) will be better explained by traditional functional groups than traits associated with plant size (PH, SM, LA), reflecting consistent functional group responses in resource addition experiments (fertilization and warming), but not in other experimental types (Dormann & Woodin, 2002).

#### 1.1.2 Does functional group composition align with post hoc trait‐based clustering of species?

We compare the species composition and explanatory power of traditional functional groups with two statistically derived, trait‐based clustering approaches, which represent optimal grouping of species within multivariate trait‐space. Given that traditional functional groups were formulated using trait‐based clustering, albeit with a smaller species pool, we hypothesize that post hoc classification will produce similar species groupings to traditional functional groups. This approach directly addresses calls to compare traditional functional groups with other trait‐based classifications (Boulangeat et al., 2012; Dorrepaal, 2007; Hudson et al., 2011), and provides the first trait‐based assessment of traditional functional groups at the tundra biome scale.

## 2 MATERIALS AND METHODS

### 2.1 Tundra biome definition

In line with previous biome‐scale assessments of tundra vegetation community change, we considered the tundra biome as the vegetated regions above tree line, both at high latitude and high altitude (Bliss, Heal, & Moore, 1981; Elmendorf, Henry, Hollister, Björk, Boulanger‐Lapointe, et al., 2012). Tundra plant communities include many widely distributed common species, and functional groups are considered to be consistent across the large geographical gradients and variety of environments within the tundra (Henry & Molau, 1997).

### 2.2 Dataset

We established a database of tundra plant traits by combining 18,613 plant trait records from the TRY database (Kattge et al., 2011; Appendix B) with 37,435 records from Tundra Trait Team (TTT) contributors (Bjorkman et al., 2018a), forming the largest database of tundra plant traits compiled to date. We considered all species present at International Tundra Experiment (ITEX) and associated plots as tundra species (Bjorkman et al., 2018b; Henry & Molau, 1997; Elmendorf, Henry, Hollister, Björk, Boulanger‐Lapointe, et al., 2012). We included all available trait records for tundra species, but excluded records from manipulated locations such as experiments or botanical gardens. Of the 449 species in the ITEX dataset, 386 (86%) had trait data available. Species lacking trait data were generally rare or uncommon species unique to single sites, and on average represented <3% of total plant cover across all sites.

We combined taxonomic synonyms following The Plant List (www.theplantlist.org) to ensure consistent taxonomy across all studies. As sampling problems inevitably arise from compiling trait data from a large number of disparate studies (Jetz et al., 2016), we removed duplicate entries, obviously erroneous values (e.g., values <0), and observations more than four standard deviations from each species mean (see Bjorkman et al., 2018a for more information). For seed mass, which is prone to measurement error due to the small masses involved and large variation within individuals (Pérez‐Harguindeguy et al., 2013), we manually checked values more than three standard deviations from each species’ mean and removed values that had clear measurement or transcription error.

### 2.3 Trait selection

We selected six plant traits for analyses: plant height (maximum measured height), seed mass (dry mass), leaf area per leaf (fresh leaf area), specific leaf area (ratio of fresh leaf area to dry leaf mass), leaf dry matter content (ratio of leaf dry mass to fresh leaf mass) and leaf nitrogen (nitrogen per unit leaf dry mass). A total of 295 species had data available for all six traits. A review of the ecological associations of each trait can be found in Díaz et al. (2016). We additionally tested two traits with low data availability, stem density (ratio of stem dry mass to fresh stem volume) and leaf life span. These traits align with key characteristics of functional groups, but are rarely measured for tundra species (Supporting Information Table S1). We log‐transformed trait values to account for log‐normal distributions, standardized between 0 and 1 using variance scaling, and aggregated traits at the species level to allow multivariate comparison among species and different units of measurement. Within‐species variation cannot be captured using this approach, but is assumed not to contribute to a large proportion of trait variation at the biome scale (Siefert et al., 2015). However, we also re‐ran analysis using the 25th and 75th percentile of species‐level trait data, representing the lowest and highest quarter of trait values for each species, respectively, to test whether results were altered by within‐species variation in the dataset as a whole.

### 2.4 Trait variation explained by functional group

We assigned species to four functional groups—evergreen shrubs, deciduous shrubs, graminoids and forbs—based on previous classification of ITEX species (Elmendorf, Henry, Hollister, Björk, Boulanger‐Lapointe, et al., 2012). We also examined two more detailed functional group classifications: (a) a six‐group classification separating graminoids into grasses, sedges and rushes and a (b) seven‐group classification further separating evergreen and deciduous shrubs into dwarf and tall shrubs. To examine the distribution of individual traits within and among functional groups, we plotted the distribution of species‐level mean traits for each of the six plant traits studied and tested the significance of distributions using pairwise Wilcoxon signed‐rank tests. To visualize multivariate trait distributions and examine the weighting of different traits, we performed principal components analysis (PCA) on multivariate trait distributions using the “prcomp” function in the R “stats” package, and plotted the first two component axes. We conducted PERMANOVA analysis to test the significance of and variance explained by functional groups to estimate how well traditional functional groups represent trait characteristics. We used Euclidian distance with 999 permutations for the combination of all six traits using the “adonis” function in the R package “vegan” (Oksanen et al., 2013).

We performed all analyses at the biome scale using all trait data, encompassing 1,333 unique georeferenced locations and non‐georeferenced trait data for tundra species. To examine if functional group significance was affected by species composition, we also conducted analyses at three unique geographical locations: Abisko (northern Sweden, 68°N, 18°E, 98 species available) representing European subarctic tundra, Davos (the Swiss Alps, 47°N, 10°E, 67 species available) representing European alpine tundra, and Qikiqtaruk‐Herschel Island (northern Canada, 69°N, −139°E, 16 species available) representing North American arctic tundra. We chose these sites to represent variation in geography and species richness across the tundra. We also repeated all analyses using a subset of only georeferenced trait data collected north of 60°N to examine if findings were influenced by environmental variation across collection locations.

To examine if the variation explained by functional groups was dependent on the traits included in analysis, we repeated PERMANOVA analysis for every possible multivariate combination of traits. This enabled us to test whether particular trait combinations were well differentiated by functional groups. We also differentiated between size‐related and economic traits, reflecting the two major dimensions of trait variation amongst global plant species (Díaz et al., 2016). As some traits were available for more species than others, resulting in unequal sample sizes among different trait combinations, we randomly selected 295 species (the minimum number of species for which all six traits were available) for each trait combination and calculated the mean variance explained over 999 replications for each combination.

### 2.5 Comparison with post hoc classifications

We compared the species composition and explanatory power of functional groups to post hoc species classifications created using statistical clustering of species‐level plant traits. We grouped species using two contrasting clustering approaches, k‐means clustering (k‐means) and hierarchical agglomerative clustering (HCA). K‐means clustering employs a top‐down approach, assigning species to groups based on multivariate distance from group means (Ding & He, 2004). Hierarchical agglomerative clustering employs a bottom‐up approach, iteratively combining groups with similar traits (Lukasová, 1979). We performed clustering using the R package “vegan” and selected a four‐cluster solution for both methods to correspond with the number of functional groups. When testing alternative six‐ and seven‐functional group classifications we selected six‐cluster and seven‐cluster solutions, respectively. For HCA clustering, we used Euclidian distance and Ward’s criterion to measure linkage. We compared differences in species composition between post hoc trait‐based classifications and traditional functional groups by calculating the maximum possible number of consistently categorized species amongst grouping methods. We also estimated the relative abundance of consistently grouped species within the ITEX database (Elmendorf, Henry, Hollister, Björk, Boulanger‐Lapointe, et al., 2012, (Polar Data Catalogue; CCIN 10786)) using the most recent year for all plots and aggregating at the site level.

Finally, we repeated PERMANOVA analysis for post hoc trait‐based classifications and examined the variance explained by groups for all traits, for only size‐related and for only economic traits. This enabled us to: (a) test the variation remaining unexplained when using post hoc classification of species, and thus (b) test the explanatory power of traditional functional groups compared to optimal four‐group clustering of species, acknowledging that it is unlikely that all trait variation will be explained, and (c) examine whether post hoc trait‐based classifications could differentiate between axes of trait variation.

All analyses were conducted in R version 3.3.2 (R Core Team, 2017). Trait data have been submitted to the TRY database (https://www.try-db.org) and are publicly available at https://github.com/TundraTraitTeam/TraitHub. Code is available at https://github.com/hjdthomas/Tundra_functional_groups

## 3 RESULTS

### 3.1 Trait variation explained by traditional functional groups

**Figure 2:**
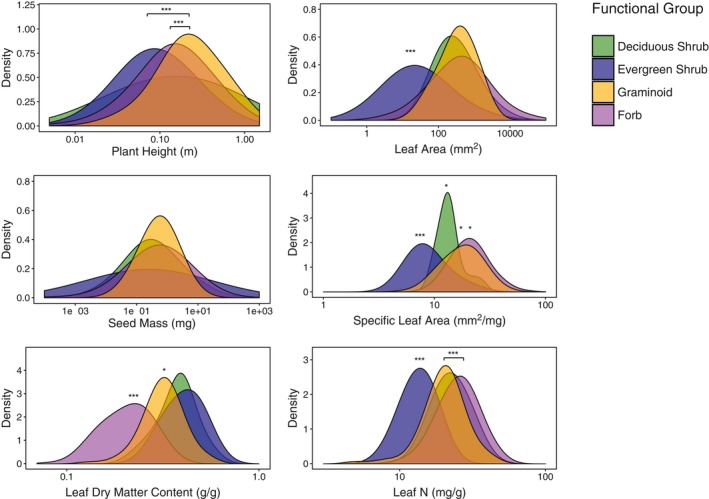
Smoothed distribution of species‐level traits represented by the four traditional tundra plant functional groups. Distributions are based on species‐level mean traits for the 295 tundra species for which data are available for all six plant traits of interest. Trait values are presented on the *x* axis in untransformed units on a log scale. Significance of distributions is indicated by symbols (pairwise Wilcoxon rank sum test; * = *p* < 0.05; ** = *p* < 0.01, *** = *p* < 0.001). Pairs of traits that are significantly different from each other, but not different from other functional groups, are indicated by black bars connecting the centre of those two distributions

**Figure 3:**
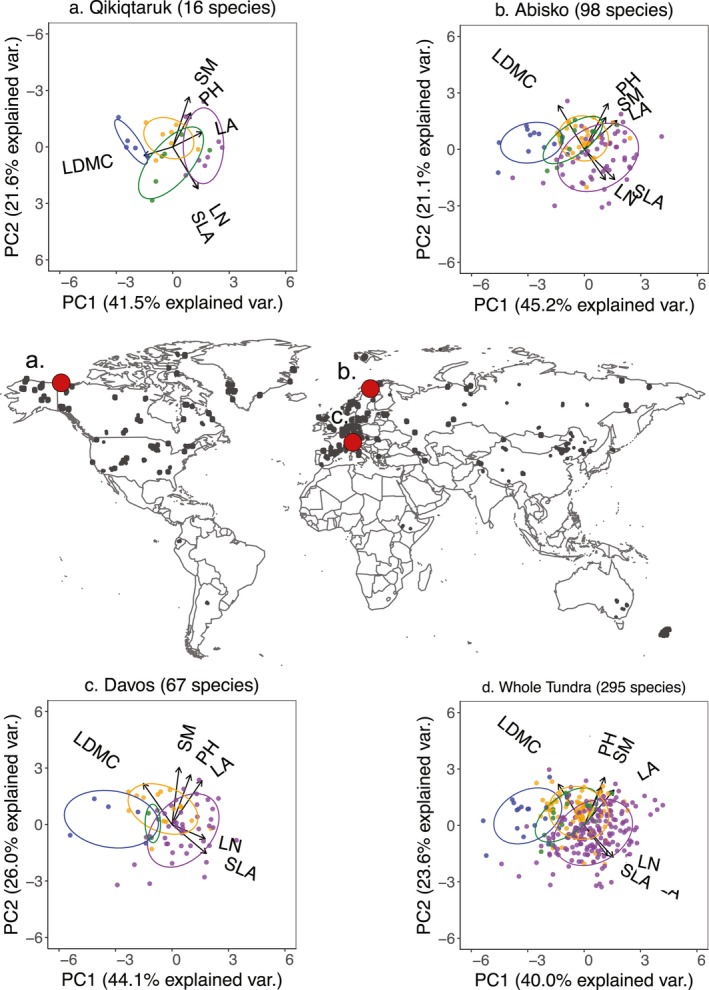
Distribution of tundra species in trait space. Inset plots indicate principal components analysis (PCA) multivariate distribution of six plant traits for three tundra sites, (a) Qikiqtaruk, (b) Abisko (c) Davos, and for (d) the whole tundra biome. Trait space was defined based on plant height (PH), seed mass (SM), leaf area (LA), specific leaf area (SLA), leaf dry matter content (LDMC) and leaf nitrogen content (LN). Individual species are represented by points and functional groups by point colour (blue = evergreen shrub, green = deciduous shrub, yellow = graminoid, purple = forb). Ellipses represent 95% confidence interval of functional group distributions. Arrows indicate direction and weighting of each trait. Georeferenced trait collection locations are indicated on the map by grey circles and modelled site locations by red circles

We found large overlap between the trait distributions of functional groups for the majority of traits examined, such that trait distributions were often not significantly different among functional groups (Figure 2, Supporting Information Figure S1). The significance of functional group distributions was strongly trait dependent, for example with significant differences among all groups for specific leaf area, but no significant differences between any groups for seed mass. Among functional groups, evergreen shrubs exhibited the most distinct differences in trait expression compared to other tundra plants, primarily driven by economic traits (Figures 2 and 3). In contrast, deciduous shrubs and graminoids exhibited largely overlapping trait distributions for many individual traits and in multivariate trait‐space.

Functional groups explained 18.5% of multivariate trait expression among species across all six traits (four‐cluster PERMANOVA, *R* ^2^ = 0.185, *p* < 0.001), and were significant both for the tundra biome and at the site level. The direction of trait weightings indicated that economic traits (SLA, LDMC, LN; greater association with PCA axis 1) and size‐related traits (PH, SM, LA; greater association with PCA axis 2) comprised distinct axes of trait variation, with functional groups primarily differentiated along the first PCA axis. The relative position of functional groups was consistent among sites, regardless of species composition or geographical location (Figure 3).

**Figure 4:**
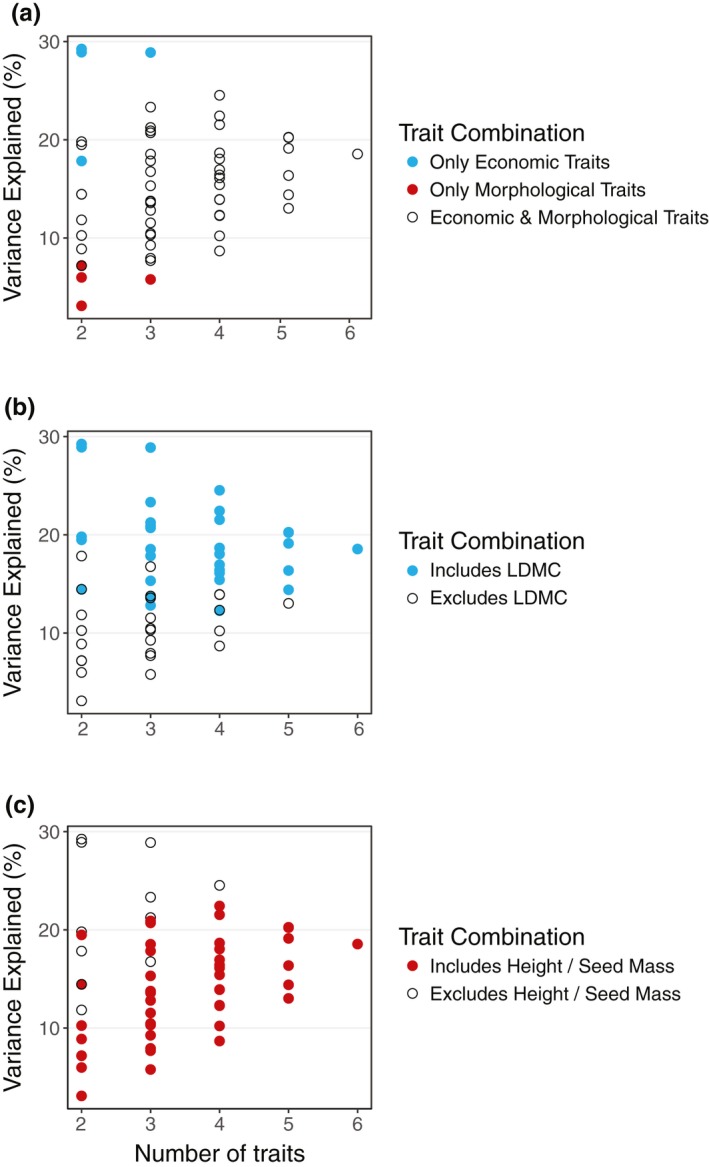
Trait variation explained by functional groups for all possible trait combinations. Functional groups best explained combinations of (a) only economic traits, or (b) those containing leaf dry matter content (LDMC), and worst explained combinations of only morphological traits or (c) those containing plant height or seed mass. Points indicate the mean variance explained (PERMANOVA *R* ^2^) by functional groups and coloured to visualize the importance of different trait combinations

The explanatory power of functional groups was strongly dependent on the traits included in the analysis. Trait combinations including only economic traits (SLA, LN, LDMC) were better explained by functional groups than size‐related traits (PH, SM, LA), regardless of the number of traits included in analysis (Figure 4a). This was largely driven by LDMC, as combinations containing this trait were best explained by functional groups (Figure 4b). In contrast, trait combinations containing PH or SM were comparatively poorly explained by functional groups (Figure 4c). Inclusion of leaf life span and stem density traits reduced data availability by over 80% (Supporting Information Table S1) but improved the explanatory power of groups from 19% to 55% and 41%, respectively. This improvement was driven by economic differences, and primarily differentiated shrubs (wood density) or evergreen shrubs (leaf life span) from other groups (Supporting Information Figure S4).

### 3.2 Comparison of post hoc trait‐based classifications with functional groups

**Figure 5:**
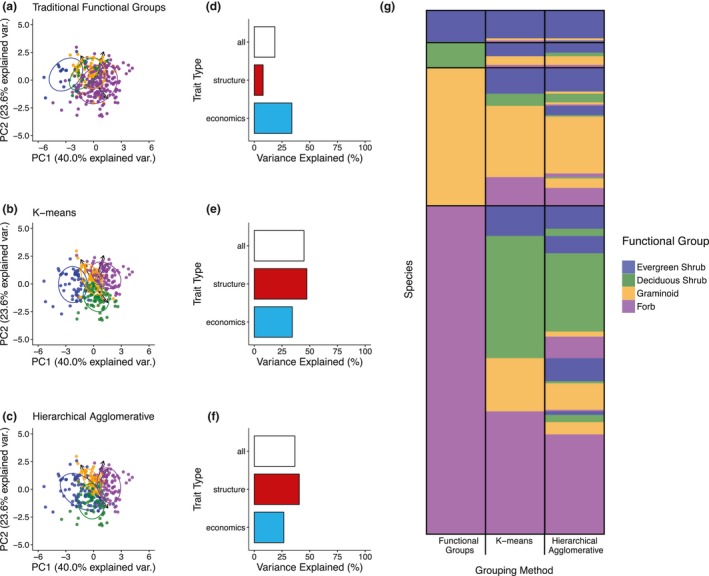
Comparison of group structure, trait variation explained, and group composition between traditional functional groups and post hoc classifications. (a–c) principal components analysis (PCA) visualization of species clusters as defined by (a) traditional functional groups, (b) k‐means clustering, and (c) hierarchical‐agglomerative clustering (HCA). Species are indicated by points and group distribution by ellipses. Colours indicate groups (dark blue = evergreen shrub, green = deciduous shrub, yellow = graminoid, purple = forb). Post hoc classifications are matched with functional groups based on maximum species correspondence between grouping methods, such that each post hoc classification corresponds with a traditional functional group. Post hoc groups approximately represent (i) tall species with large leaves and seeds (purple), (ii) mid‐sized species with economically acquisitive strategies (yellow), (iii) small species with economically acquisitive strategies (green) and (iv) small species with economically conservative strategies (blue). (d–f) Trait variation explained by (d) traditional functional groups, (e) k‐means, and (f) hierarchical agglomerative clustering (HCA) for multivariate combinations of all six plant traits (white), size‐related traits only (red) and economic traits only (light blue). (g) Comparison of group composition across clustering methods. The stacked bars represent individual species and are ordered by traditional functional group (species order remains consistent across columns). The colour of each stacked bar represents the group to which species were assigned by each classification method (classification can change across columns). For example, a species categorized as a graminoid by traditional functional groups can be categorized in the group most corresponding to forbs by post hoc classifications

Post hoc trait‐based classification of species did not correspond well with traditional functional group composition. The four groups identified by post hoc classification were consistently located within trait‐space across clustering methods, and were differentiated by the two axes of trait variation, although more strongly by size‐related traits (Figure 5). Post hoc classifications thus represented: (a) tall species with large leaves and seeds (high PH, SM and LA), (b) mid‐sized species with economically acquisitive strategies (low LDMC, high SLA and LN), (c) small species with economically acquisitive strategies, and (d) small species with economically conservative strategies.

Forty‐two per cent of species were consistently classified between traditional functional groups and k‐mean clustering, and 43% between traditional functional groups and HCA clustering (Figure 5f, Table 1). In contrast, 74% of species were consistently classified between post hoc clustering methods. Evergreen shrubs, approximately half of graminoids and one third of forbs were largely assigned to consistent groups across the three clustering methods (Figure 5f). Deciduous shrubs showed very low correspondence between functional groups and post hoc classifications due to large trait overlap with both graminoids and forbs, but showed high correspondence between clustering methods (Table 1, Supporting Information Table S2).

**Table 1:** *Top*: Similarity in species composition between traditional functional groups and post hoc trait‐based classifications (k‐means = k‐means clustering; HCA = hierarchical agglomerative clustering), calculated as the proportion of consistently classified species out of all species. *Bottom*: Relative abundance of consistently classified species within tundra (International Tundra Experiment, ITEX) vegetation communities, calculated as the proportion of the summed abundance of consistently classified species out of the summed abundance of all species for which trait data are available across all ITEX plots

Functional group	Functional groups versus k‐means (%)	Functional groups versus HCA (%)	k‐means versus HCA (%)	All methods (%)
**Similarity between group species composition**				
All groups	42	43	74	35
Evergreen shrubs	89	94	94	89
Deciduous shrubs	0	13	87	0
Graminoids	52	51	78	42
Forbs	37	37	69	30
**Relative abundance of consistent species**				
All groups	56	59	87	51
Evergreen shrubs	99	100	99	99
Deciduous shrubs	0	21	79	0
Graminoids	74	65	84	62
Forbs	24	32	82	22

Abundant species were more likely to be consistently classified across grouping methods (Supporting Information Figure S2a), and the relative abundance of consistently classified species within tundra plant communities (51%) was greater than would be expected if all species had equal abundance (35%). Although abundant species had more available trait observations, and thus may have more representative species‐mean traits, the number of trait observations did not significantly affect whether a species was consistently classified (Supporting Information Figure S2b). Species that were consistently categorized across grouping methods occupied a distinct region of trait‐space (*p* < 0.001) and were mostly large (taller, larger leaves or larger seeds) with extreme economic traits (i.e., highly conservative or highly acquisitive species, Supporting Information Figure S2d). Inconsistently classified species had traits closer to the centre of the overall distribution of tundra species within functional trait space, suggesting that the traits of these species may be poorly represented by traditional functional groups.

Post hoc classifications explained 45% (k‐means, *R* ^2^ = 0.448, *p < *0.001) and 37% (HCA, *R* ^2^ = 0.366, *p < *0.001) of trait variation amongst tundra species, compared to 19% for traditional functional groups (Figure 5d–f). Despite derivation using all six plant traits, post hoc classifications explained greater variation in size‐related traits than traditional functional groups for both clustering methods (functional groups: *R* ^2^ = 0.080, *p* < 0.001; k‐means: *R* ^2^ = 0.474, *p* < 0.001; HCA: *R* ^2^ = 0.406, *p* < 0.001), whilst k‐means sampling also slightly better explained variation in economic traits (functional groups: *R* ^2^ = 0.339, *p* < 0.001, k‐means:* R* ^2^ = 0.343, *p* < 0.001; HCA: *R* ^2^ = 0.266, *p* < 0.001, Figure 5d–f). Our results demonstrate that unexplained trait variation does not solely arise due to aggregation of species into a small number of groups, and that functional groups have less than half the explanatory power of optimal species classification for the six most commonly collected tundra plant traits.

## 4 DISCUSSION

### 4.1 Trait variation is poorly explained by traditional functional groups

To be meaningful for ecological analyses, plant functional groups should accurately and consistently represent differences in species characteristics that underpin their environmental preferences and responses (Chapin et al., 1996). In this study, we find that traditional plant functional groups represent 19% of variation in the six most commonly measured plant traits amongst tundra species. Furthermore, the species composition of functional groups did not align well with post hoc trait‐based classification of species. Together, our findings indicate that traditional functional groups poorly represent species‐level variation in the six plant traits considered by this study, and highlight potential limitations of functional group approaches to predicting community responses to environmental change in the tundra.

Our findings support a previous trait‐based criticism of traditional functional groups in European alpine species (Körner et al., 2016), and may explain low explanatory power and contradictory responses of functional groups in previous tundra studies (Dormann & Woodin, 2002; Dorrepaal, 2007; Figure 1). Although it is possible that the tundra is unusual in the global context due to small plant growth‐forms and harsh environmental conditions, our study is in line with findings that functional groups poorly describe trait variation in tropical forests (Wright et al., 2013), temperate grasslands (Forrestel et al., 2017; Fry, Power, & Manning, 2014; Wright et al., 2006), and among certain traits at the global scale (Iversen et al., 2017; Kattge et al., 2011; Reichstein, Bahn, Mahecha, Kattge, & Baldocchi, 2014; Wright et al., 2005).

Our findings for the six most commonly measured traits in part contradict Chapin et al.’s (1996) finding that growth‐form based functional groups can be reproduced from trait information. This discrepancy could arise from the greater number of species and individual trait records represented in our study, which may increase variability within functional groups and species, or the greater number of traits included in Chapin et al. (1996). Trait variation may also be better represented by alternative classifications such as those distinguishing between tall and dwarf shrubs, or between grasses and sedges. Although alternative six‐group and seven‐group classification schemes did slightly increase the explanatory power of functional groups (from 18.5% to 21.4% and 24.9%, respectively, Supporting Information Figure S3), the overall variance explained remained low and substantially less than post hoc classifications (53.6% and 56.8%, respectively).

**Figure 6:**
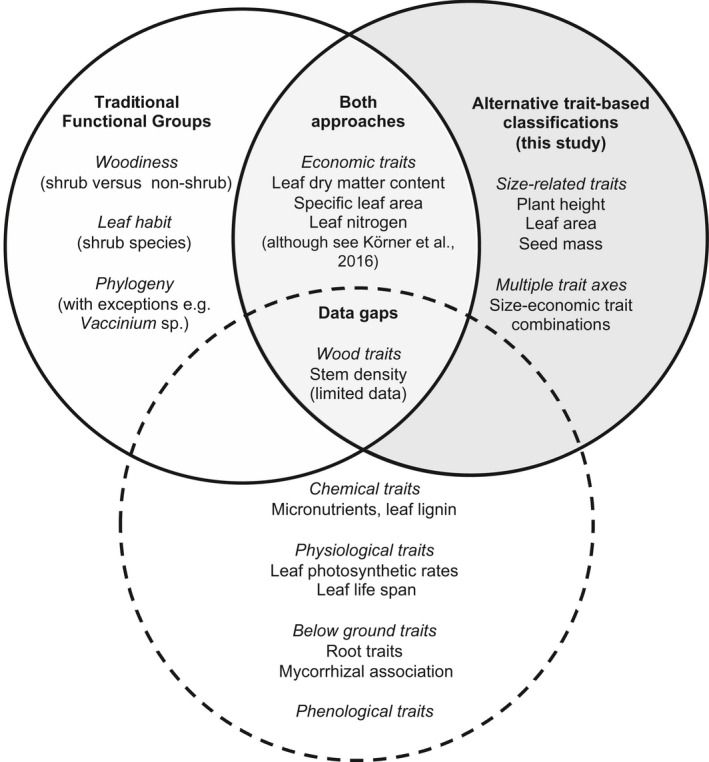
Functional groups and post hoc trait‐based classifications capture different characteristics of tundra plant communities. Solid circles enclose characteristics represented by functional groups, post hoc classifications, and by both approaches, according to the findings of this study. The dotted circle encloses the data gaps for traits that are not well represented in tundra trait databases or trait‐based analysis yet are suggested to be important in the literature (Bardgett, Mommer, & Vries, 2014; Chave et al., 2009; Cleland et al., 2012; Eckstein et al., 1999)

Low explanatory power of functional groups could also arise from the choice of traits included in analysis. The traits investigated in this study are considered critical determinants of ecological processes (Díaz et al., 2016; Pérez‐Harguindeguy et al., 2013), and represent both available tundra trait data and the focus of trait‐based research in tundra ecosystems (Bjorkman et al., 2018a). Nevertheless, we found that the explanatory power of functional groups was highly trait‐specific (Figure 4), and thus functional groups may represent differences amongst plant traits not investigated here that are nonetheless critical to ecosystem function in the tundra (Figure 6). For example, inclusion of stem density increased the explanatory power of traditional functional groups to over 50% (Supporting Information Figure S4), but reduced species representation by 80% (*n* = 53) and did not improve representation of size‐related traits.

### 4.2 Functional groups align with economic traits

Among tundra species, traditional functional groups better represented variation in economic traits (SLA, LDMC, LN) than size‐related traits (PH, SM, LA). Indeed, functional groups explained roughly equal variation in economic traits to post hoc clustering (33.5% compared to 34.3% for k‐means clustering). As such, ecosystem functions related to resource economics such as photosynthetic rate or nutrient cycling may be well represented using functional group approaches (Lavorel & Garnier, 2002). This difference may also explain why studies focusing on community responses to resource addition (Dormann & Woodin, 2002; Elmendorf, Henry, Hollister, Björk, Bjorkman, et al., 2012; Zamin et al., 2014) or litter quality (Carbognani, Petraglia, & Tomaselli, 2014; Cornelissen et al., 2007; Dorrepaal et al., 2005) find the clearest differences between functional groups.

Low representation of size‐related traits may arise due to convergence of growth forms in the tundra; all functional groups contain both comparatively large (e.g., the tall deciduous shrub *Salix glauca* or forb *Chamaenerion angustifolium*) and comparatively small (eg, the dwarf deciduous shrub *Salix polaris* or forb *Saxifraga bryoides*) species. As a result, functional groups may poorly represent ecosystem functions or properties relating to size‐related traits, such as albedo, carbon storage, seed dispersal or competitive ability (Lavorel & Garnier, 2002; Loranty, Goetz, & Beck, 2011; Westoby, Falster, Moles, Vesk, & Wright, 2002). Such properties are implicated as key drivers of community‐level vegetation change in the tundra (Kaarlejärvi, Eskelinen, & Olofsson, 2017; Mekonnen et al., 2018). Functional group classifications that explicitly recognize morphological characteristics, such as distinguishing between tall and dwarf shrubs (Elmendorf, Henry, Hollister, Björk, Boulanger‐Lapointe, et al., 2012; Vowles et al., 2017), may better characterize differences in trait expression, although we found limited evidence for this (Supporting Information Figure S3). As such, post hoc classification of species or direct use of trait data may identify differences amongst size‐related traits, and associated drivers of community change and ecosystem function, that are obscured by variation within traditional functional groups (Matesanz, Escudero, & Fernando, 2009).

### 4.3 Trait‐based approaches as an alternative to functional groups

Our findings contribute to growing support for the use of trait‐based approaches as an alternative to functional groups within ecological research and earth system modelling. Trait‐based approaches include post hoc grouping of species according to common traits (Suding et al., 2008), common responses to environmental conditions (Cornwell & Ackerly, 2010) or common effects on ecosystem processes (Cornwell et al., 2008; Laughlin, 2011), as well as direct use of trait data in analysis (McGill et al., 2006). In this study, post hoc classifications explained more than twice as much trait variation as functional groups, and were distinguished along two global axes of trait variation (Díaz et al., 2016), representing large versus small species, and economically “fast” versus “slow” species (Díaz et al., 2016; Reich, 2014). Post hoc classifications thus better captured the multidimensionality of trait variation compared to traditional groupings (Maire, Grenouillet, Brosse, & Villéger, 2015), and produced relatively robust species groupings across the two clustering methods.

Post hoc approaches have nevertheless been criticized on the basis of inconsistencies across methodologies and ecological communities (Dyer, Goldberg, Turkington, & Sayre, 2001; Fry et al., 2014), and could be biased towards representing rarer species with more extreme traits. In this study, functional groups better represented differences amongst more abundant species (Table 1), and thus may capture community‐level characteristics even if representation of differences amongst individual species is low. Species that were consistently categorized (Supporting Information Table S3) possessed similar traits including a larger structure (tall with large leaves and seeds) and either highly conservative or acquisitive resource economic traits. However, some species that were inconsistently classified, notably deciduous shrubs such as *Betula nana* and graminoids such as *Agrostis spp*., have demonstrated the greatest vegetation responses at many tundra sites (Bret‐Harte et al., 2001; Venn, Pickering, & Green, 2014), suggesting that traditional functional groups may obscure some important trait characteristics associated with vegetation change (Saccone et al., 2017).

### 4.4 Underpinning assumptions

The findings of this study are based on several key assumptions. First, we assume that the species for which trait data are available are representative of all tundra species. Species lacking trait data are often rare (low abundance) or endemic (occur at few sites). The data gap for these missing species could represent unusual trait combinations not easily captured by trait‐based classification (Sandel et al., 2015). We also do not examine mosses and lichens, which play an important role in ecosystem function in the tundra (Turetsky, Mack, Hollingsworth, & Harden, 2010). Nevertheless, the species included in this study reflect the majority of tundra plant biomass and include the species known to be most rapidly responding to climate change (Elmendorf, Henry, Hollister, Björk, Boulanger‐Lapointe, et al., 2012).

Second, we assume that plant traits are meaningful predictors of species’ responses to environmental dynamics or effects on ecosystem function. In this study, we do not examine whether traits or alternative trait‐based classifications better predict community dynamics than functional groups. Traditional functional groups may better predict certain ecological dynamics than trait‐based approaches as they integrate multiple measured and unmeasured traits across plant organs, ecological strategy, and life cycle (Grime et al., 1997). Nevertheless, there is widespread evidence to support trait‐based approaches to modelling ecosystem dynamics (Suding et al., 2008; Violle & Jiang, 2009; Cornwell & Ackerly, 2010; Soudzilovskaia et al., 2013, but see Clark, 2016). Single traits, such as plant height, have also predicted vegetation responses to change that are obscured within traditional functional groups (Elmendorf, Henry, Hollister, Björk, Boulanger‐Lapointe, et al., 2012). Continuing to assess the extent to which trait‐based approaches can meaningfully describe and predict ecosystem processes therefore remains an essential research focus (McGill et al., 2006). Differentiating community responses or ecosystem processes using post hoc trait‐based classifications would provide a direct test of this question, and could offer valuable insight into the relative importance of different traits for prediction and modelling.

Third, we assume that the majority of trait variation occurs among species. Should large trait variation occur *within* species this could invalidate species‐level clustering (Shipley et al., 2016; Violle et al., 2012). The species considered in this study have large geographical ranges, encompassing both Arctic and alpine tundra, and nontundra locations. However, our findings are robust when using individual trait‐data (Supporting Information Figure S1), across site‐specific species assemblages (Figure 3), for the 25th and 75th percentile of species‐level trait data (Supporting Information Figure S5), and for only trait collection locations north of 60°N (Supporting Information Figures S6–S9). Furthermore, most studies have found within‐species variation to be small compared to among‐species variation (Anderegg et al., 2018; Kattge et al., 2011; Siefert et al., 2015), including in the tundra biome (Thomas et al., in prep, manuscript available upon request). Nevertheless, within‐species trait variation may be an important driver of community change, particularly at small spatial scales, and may explain highly individualistic species responses to change (Hollister et al., 2005). Thus, we advocate that studies should recognize and account for the extent of trait variation within communities.

Finally, attempts to classify species into functional groups may be impossible if trait expression or species response is dependent upon environmental and ecological context (Dorrepaal, 2007; Laughlin & Messier, 2015). Group classifications and even growth strategies may change depending on resource availability (Bret‐Harte et al., 2001), such that division into discrete classifications may obscure the variability inherent to natural environments (Westoby & Wright, 2006). Although differences between functional groups were statistically significant in this study, the majority of trait variation was not explained by classifications, whether using traditional functional groups (81% of variance unexplained) or post hoc classification (55% of variance unexplained). We, therefore, join those who advocate that ecological analyses should continue to move towards incorporating explicitly trait‐based approaches, focusing on traits themselves as the fundamental units of analysis (Laughlin, 2014; McGill et al., 2006; Violle & Jiang, 2009; Weiher et al., 2011; Westoby & Wright, 2006).

### 4.5 Future priorities

Our findings suggest that new trait data collection campaigns should focus on traits that distinguish among ecological strategies and responses to changing growing conditions. Whilst existing trait records have been informed by standardized protocols and contemporary research priorities (Cornelissen et al., 2003; Pérez‐Harguindeguy et al., 2013), these have tended to focus on easily measurable leaf traits. Future trait collection campaigns should therefore focus on ecologically important traits for which we have few records, including chemical and physiological traits (Eckstein, Karlsson, & Weih, 1999), and whole‐plant measurements, incorporating stem (Chave et al., 2009) and belowground (Iversen et al., 2015) characteristics. Finally, phenological traits such as leaf out or flowering time are rarely integrated into wider trait‐based approaches, yet may be critical to predicting ecological responses, particularly in a warming tundra (Cleland et al., 2012).

## 5 CONCLUSION

In this study, we demonstrate that traditional plant functional groups poorly represent differences in the six most commonly measured plant traits among tundra vascular plant species. Although functional groups were statistically distinct and consistent among sites, they explained only 19% of overall trait variation and primarily differentiated between resource economic traits rather than size‐related traits. Post hoc trait‐based classification of species did not align with functional group classification, but produced robust alternative groupings that aligned with two global axes of trait variation. Together, our findings indicate that traditional functional groups may not characterize trait variation within tundra vegetation communities, particularly among size‐related traits. We therefore argue that: (a) traditional functional groups should be used with caution when testing ecological responses or ecosystem functions associated with size‐related traits; (b) functional group approaches require sufficient species and trait measurements to capture variation within groups, within species and among traits; and (c) the use of alternative classifications based on trait expression, or direct use of underlying trait data, could provide new insights for predicting vegetation change and ecosystem processes in response to global drivers of environmental change.

## AUTHOR CONTRIBUTIONS

HT and IMS conceived the study. HT performed statistical analysis with additional input from IMS and AB. HT wrote the manuscript with input from IMS and AB with contributions from all authors. AB compiled the TTT database with assistance from IMS, SE and AB led the sTundra working group. IMS supervised HT and acquired funding for the project. Authorship order was based on total contribution to the manuscript for the first four authors (see above), and then (a) input from the sTundra working group and contribution to TTT (alphabetical), (b) input from the sTundra working group and contribution to TRY (alphabetical), (c) input from the sTundra working group only (alphabetical), (d) contribution to the TTT database (alphabetical), and (e) contribution to TRY (alphabetical).

## BIOSKETCH

This work was led by **Haydn J. D. Thomas** as part of the sTUNDRA working group. Haydn is a plant ecologist interested in how the tundra biome is changing. His work primarily focuses on whether, and how, plant traits can be used to predict change to plant communities and ecosystem function in tundra ecosystems. The sTUNDRA working group is an international collaboration exploring tundra vegetation and trait change across biome‐scale climate gradients and over three decades of ecological monitoring led by Isla H. Myers‐Smith, Anne D. Bjorkman and Sarah C. Elmendorf. Trait data were provided by the “Tundra Trait Team”, compiled by Anne D. Bjorkman. Additional trait data were provided by TRY, a network of vegetation scientists headed by Future Earth and the Max Planck Institute for Biogeochemistry, providing a global archive of curated plant traits.

## Data Availability

Trait data have been submitted to the TRY database (https://www.try-db.org) and are publicly available at https://github.com/TundraTraitTeam/TraitHub. Composition data are available in the Polar Data Catalogue (https://www.polardata.ca/ CCN 10786). Code is available at https://github.com/hjdthomas/Tundra_functional_groups
