# Supplementary material for: Traditional plant functional groups explain variation in economic but not size‐related traits across the tundra biome
Source: Glob Ecol Biogeogr. 2018 Nov 16;28(2):78–95. doi: 10.1111/geb.12783 (PMC6472633; doi:10.1111/geb.12783)
Supplement: Supplementary file 1 [file GEB-28-78-s001.pdf]

Traditional plant functional groups explain variation in economic but not size-related traits across the tundra biome

Supplementary Materials

Below are all supplementary tables and figures supporting the analyses set out in 'Traditional plant functional groups explain variation in economic but not size-related traits across the tundra biome'.

Table S1 Number of trait observations and species with available trait data for the six main traits (plant height, specific leaf area, leaf dry matter content, leaf nitrogen, seed mass) and two supplementary traits (stem specific density, leaf lifespan) used in analysis.

	All trait observations		Only species with trait data for all traits	
**Trait name**	**Observations**	**Species**	**Observations**	**Species**
Plant height	26,448	742	19,272	295
Specific leaf area	15,406	562	12,517	295
Leaf dry matter content	11,691	473	9,376	295
Leaf nitrogen	6,352	471	5,342	295
Seed mass	4,230	637	3,029	295
Stem specific density	1,214	66	1,003	53
Leaf lifespan	237	129	190	90

Table S2 Similarity in species composition between traditional functional groups and trait-based classifications (*k-means* = k-means clustering; *HCA* = hierarchical agglomerative clustering), calculated as the proportion of consistently classified species out of all species. Post-hoc groups were matched to functional groups based on the maximum correspondence of each individual functional group, rather than based on overall correspondence across all functional groups as in the main analysis. Any changes to similarity using these grouping are indicated in bold, with similarities for groupings used in main analysis indicated in brackets. Only deciduous shrub and graminoids species changed between grouping approaches. Maximising the deciduous shrub grouping using this alternative approach resulted in an increase in correspondence for deciduous shrubs but an overall decrease across all groups.

Functional Group	Functional groups vs. K-means	Functional groups vs. HCA	K-means vs. HCA	All Methods
**Similarity between group species composition – Deciduous shrubs maximised**				
All groups	**33%** (42%)	**33%** (43%)	74% (74%)	**28%** (35%)
Evergreen shrubs	89% (89%)	94% (94%)	94% (94%)	89% (89%)
Deciduous shrubs	**33%** (0%)	**33%** (13%)	87% (87%)	**33%** (0%)
Graminoids	**9%** (52%)	**9%** (51%)	78% (78%)	**6%** (42%)
Forbs	37% (37%)	37% (37%)	69% (69%)	30% (30%)

Table S3 List of species that are consistently categorized to corresponding groups (104 out of 295) among traditional plant functional groups, k-means clustering, and hierarchical agglomerative clustering.

Species	Functional Group	Species	Functional Group
Andromeda polifolia	Ever. Shrub	*Anemone narcissiflora*	Forb
Calluna vulgaris	Ever. Shrub	*Anemone nemorosa*	Forb
Cassiope tetragona	Ever. Shrub	*Angelica archangelica*	Forb
Diapensia lapponica	Ever. Shrub	*Anthriscus sylvestris*	Forb
Dryas integrifolia	Ever. Shrub	*Anthyllis vulneraria*	Forb
Dryas octopetala	Ever. Shrub	*Arnica montana*	Forb
Empetrum nigrum	Ever. Shrub	*Astragalus frigidus*	Forb
Harrimanella hypnoides	Ever. Shrub	*Athyrium filix-femina*	Forb
Ledum palustre	Ever. Shrub	*Biscutella laevigata*	Forb
Linnaea borealis	Ever. Shrub	*Caltha palustris*	Forb
Loiseleuria procumbens	Ever. Shrub	*Carlina acaulis*	Forb
Phyllodoce caerulea	Ever. Shrub	*Carum carvi*	Forb
Rhododendron lapponicum	Ever. Shrub	*Crepis paludosa*	Forb
Thymus praecox	Ever. Shrub	*Filipendula ulmaria*	Forb
Vaccinium oxycoccos	Ever. Shrub	*Gentiana purpurea*	Forb
Vaccinium vitis-idaea	Ever. Shrub	*Geranium gymnocaulon*	Forb
Anthoxanthum odoratum	Graminoid	*Geranium sylvaticum*	Forb
Arctagrostis latifolia	Graminoid	*Geum rivale*	Forb
Blysmus compressus	Graminoid	*Hedysarum caucasicum*	Forb
Briza media	Graminoid	*Hieracium laevigatum*	Forb
Calamagrostis canadensis	Graminoid	*Hieracium prenanthoides*	Forb
Calamagrostis purpurea	Graminoid	*Hieracium umbellatum*	Forb
Calamagrostis villosa	Graminoid	*Lactuca alpina*	Forb
Carex aquatilis	Graminoid	*Leontodon hispidus*	Forb
Carex atrata	Graminoid	*Lomelosia caucasica*	Forb
Carex canescens	Graminoid	*Lupinus arcticus*	Forb
Carex caryophyllea	Graminoid	*Melampyrum pratense*	Forb

Carex flacca	Graminoid	*Melampyrum sylvaticum*	Forb
Carex flava	Graminoid	*Menyanthes trifoliata*	Forb
Carex montana	Graminoid	*Persicaria bistorta*	Forb
Carex nigra	Graminoid	*Petasites frigidus*	Forb
Carex pilulifera	Graminoid	*Peucedanum ostruthium*	Forb
Carex saxatilis	Graminoid	*Pimpinella major*	Forb
Carex sempervirens	Graminoid	*Plantago atrata*	Forb
Carex umbrosa	Graminoid	*Potentilla anserina*	Forb
Deschampsia cespitosa	Graminoid	*Prunella vulgaris*	Forb
Eriophorum angustifolium	Graminoid	*Pulsatilla aurea*	Forb
Eriophorum scheuchzeri	Graminoid	*Ranunculus acris*	Forb
Eriophorum vaginatum	Graminoid	*Ranunculus montanus*	Forb
Festuca rubra	Graminoid	*Ranunculus trichophyllus*	Forb
Festuca varia	Graminoid	*Rhinanthus minor*	Forb
Helictotrichon versicolor	Graminoid	*Rubus chamaemorus*	Forb
Hierochloe alpina	Graminoid	*Rumex acetosa*	Forb
Phleum alpinum	Graminoid	*Rumex alpestris*	Forb
Poa alpina	Graminoid	*Rumex aquaticus*	Forb
Poa pratensis	Graminoid	*Silene dioica*	Forb
Poa trivialis	Graminoid	*Silene vulgaris*	Forb
Trisetum flavescens	Graminoid	*Taraxacum campylodes*	Forb
Adenostyles alpina	Forb	*Trifolium pratense*	Forb
Ajuga reptans	Forb	*Trollius europaeus*	Forb
Alchemilla xanthochlora	Forb	*Veratrum album*	Forb
Anemone alpina	Forb	*Vicia cracca*	Forb

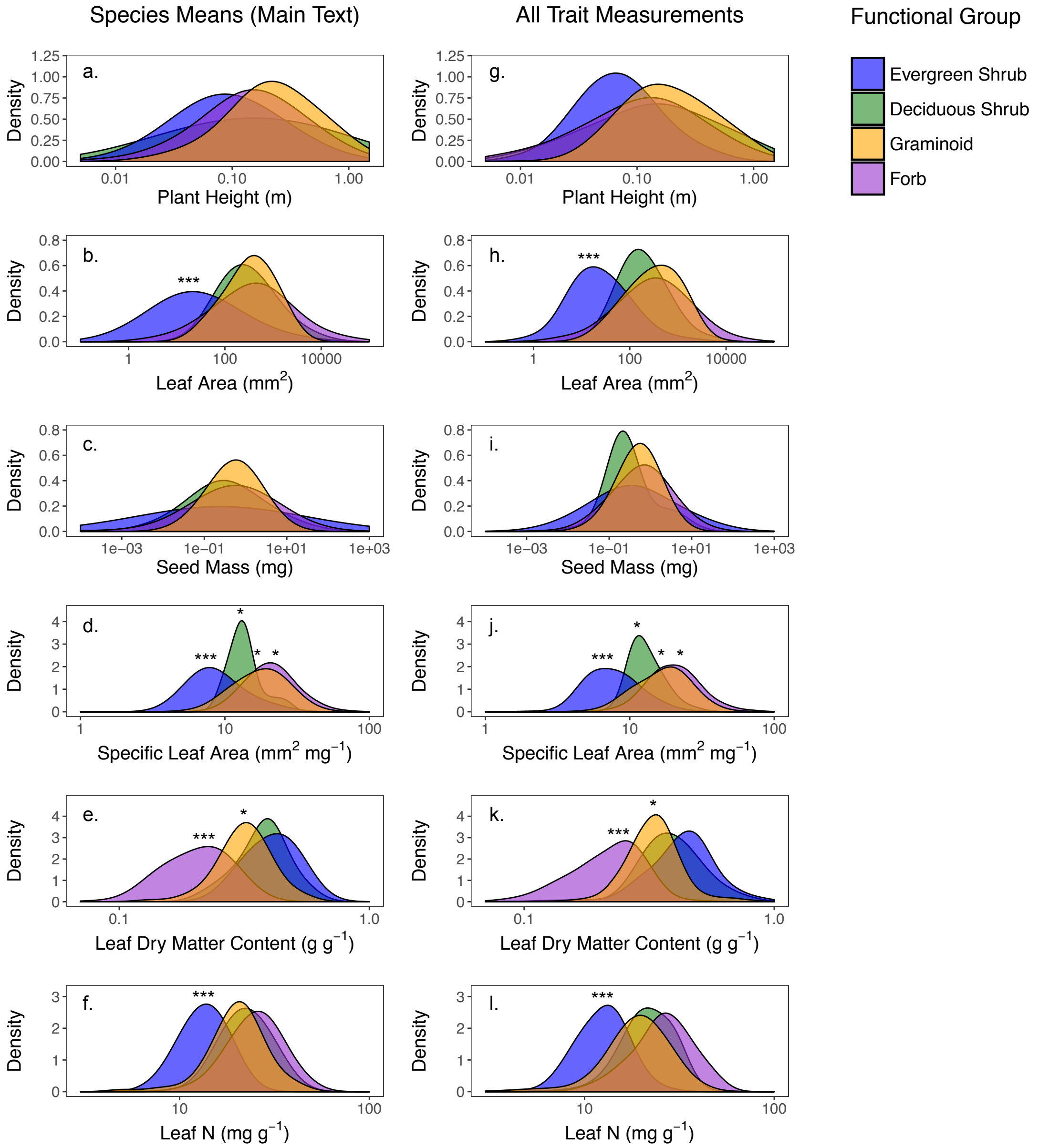

Figure S1 Distribution of tundra plant traits represented by the four traditional tundra plant functional groups. Distributions in panels (**a-f**) are based on species-level means for the 295 tundra species for which data are available , as presented in Figure 2 in the main text. Distributions in panels (**g-l**) are based on all available trait data for the 295 tundra species for which data are available for all six plant traits of interest. The use of all trait data accounts for within-species trait variation within functional groups, but is biased by species with greater availability of trait data for some species. Trait values are presented on the x axis in untransformed units on a log scale.

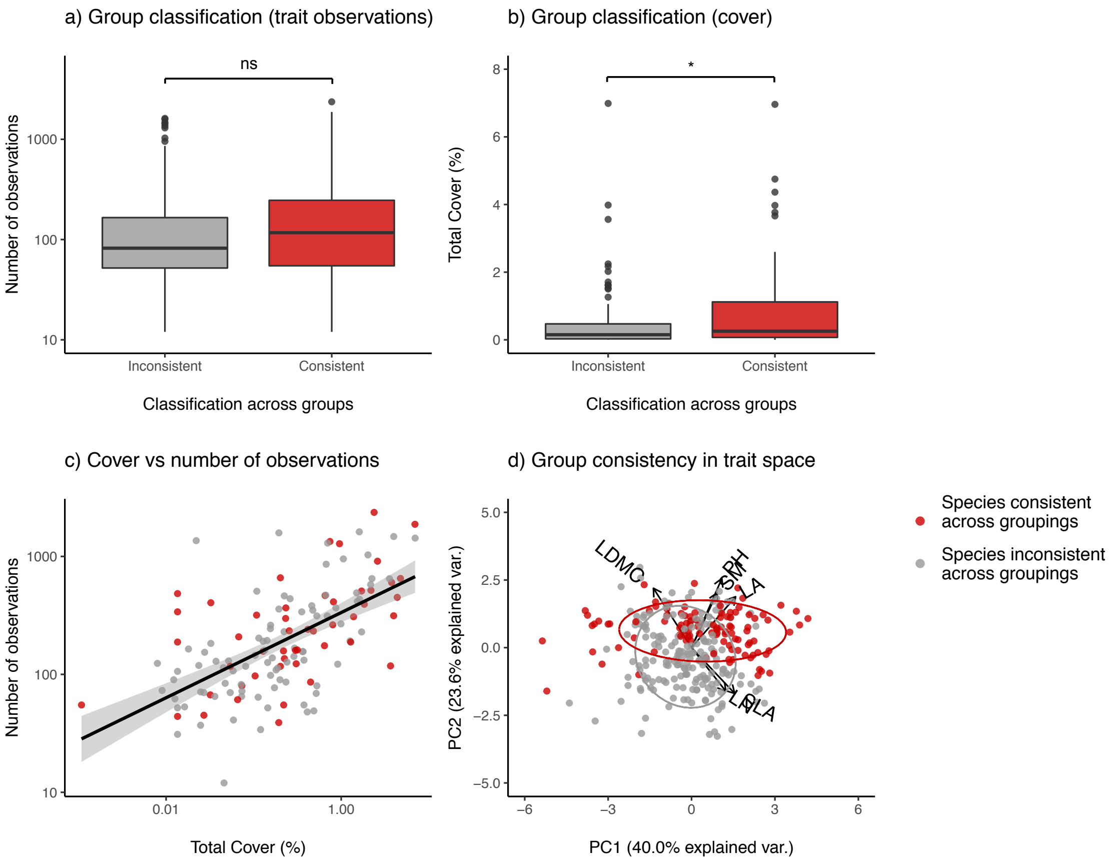

Figure S2 Abundance, but not number of observations increases likelihood that species will be consistently classified across the three sampling methods (functional groups, k-means clustering, hierarchical agglomerative clustering). Plant traits represented are plant height (PH), leaf area (LA), seed mass (SM), specific leaf area (SLA), leaf dry matter content (LDMC) and lean nitrogen per unit mass (LN). **a**) Number of trait observations for species that were consistently and inconsistently classified across clustering methods. Differences are not statistically significant (Wilcoxon test, $P = 0.11$). **b**) Relative abundance of species that were consistently and inconsistently classified across clustering methods. Differences are statistically significant (Wilcoxon test, $P = 0.02$). **c**) Relationship between number of trait observations and relative abundance of species. Point colours indicate if species were consistently classified. Line indicates linear model fit (LM, stats), and shaded area the 95% confidence intervals. **d**) Multivariate distribution of six plant traits for tundra

species, indicating species that were consistently classified across grouping methods. Species that were consistently classified (red points, 104 out of 295 species) occupied a significantly different region of trait-space (PERMANOVA, $P < 0.001$) and tended to have larger growth forms and more extreme economic traits (highly conservative or highly acquisitive). Inconsistently classified species (grey points) tended to be located closer towards the centre of the overall tundra trait distribution.

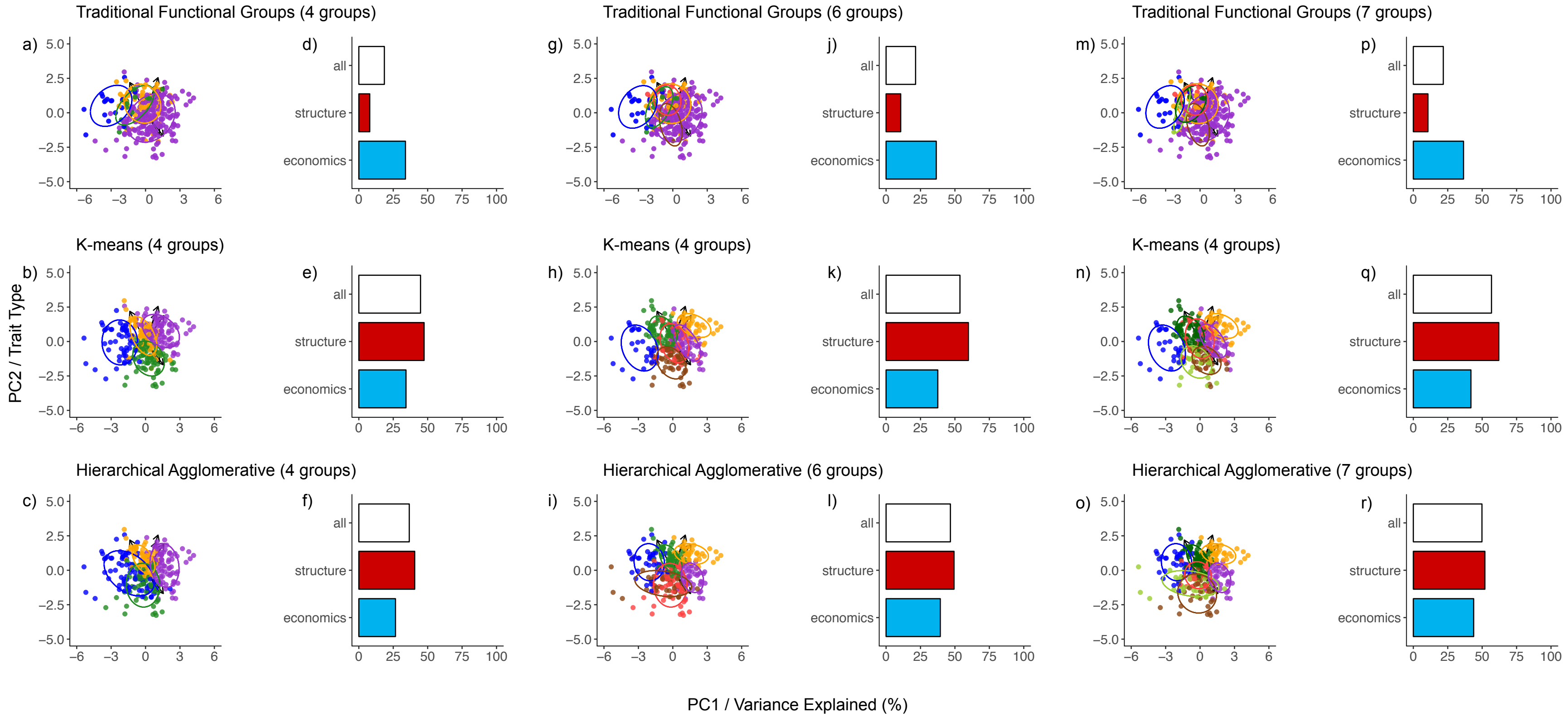

Figure S3 Alternative classification schemes increase the trait variation explained by functional groups, but in line with expectations resulting from an increased number of groups. **a-c**) Clustering of species in multivariate trait-space according to **a**) the four-group classification in the

main analysis, **b**) four-group k-means clustering, and **c**) four-group hierarchical-agglomerative sampling. **d-f**) variance explained by four-group clusters for all traits (white), only size-related traits (red) and only economic traits (blue). **g-i**) Clustering of species in multivariate trait-space according to **g**) a six-group functional group classification (evergreen shrubs (blue), deciduous shrubs (green), grasses (orange), sedges (red), rushes (brown), forbs (purple)), **h**) six-group k-means clustering, and **i**) six-group hierarchical-agglomerative sampling. **j-l**) variance explained by six-group clusters for all traits, only size-related traits, and only economic traits. **m-o**) Clustering of species in multivariate trait-space according to **m**) a seven-group functional group classification (dwarf evergreen shrubs (blue), dwarf deciduous shrubs (light green), tall deciduous shrubs (dark green), grasses (orange), sedges (red), rushes (brown), forbs (purple)). We classified shrubs with a mean height greater than 30cm as tall shrubs; there were no tall evergreen shrubs with available trait data for all six traits according to this classification. **n**) seven-group k-means clustering, and **o**) seven-group hierarchical-agglomerative sampling. **p-r**) variance explained by seven-group clusters for all traits, only size-related traits, and only economic traits. Functional space was defined based on plant height (PH), seed mass (SM), leaf area (LA), specific leaf area (SLA), leaf dry matter content (LDMC) and leaf nitrogen content (LN). Ellipses represent 95% confidence interval of functional group distributions. Arrows indicate direction and weighting of each trait.

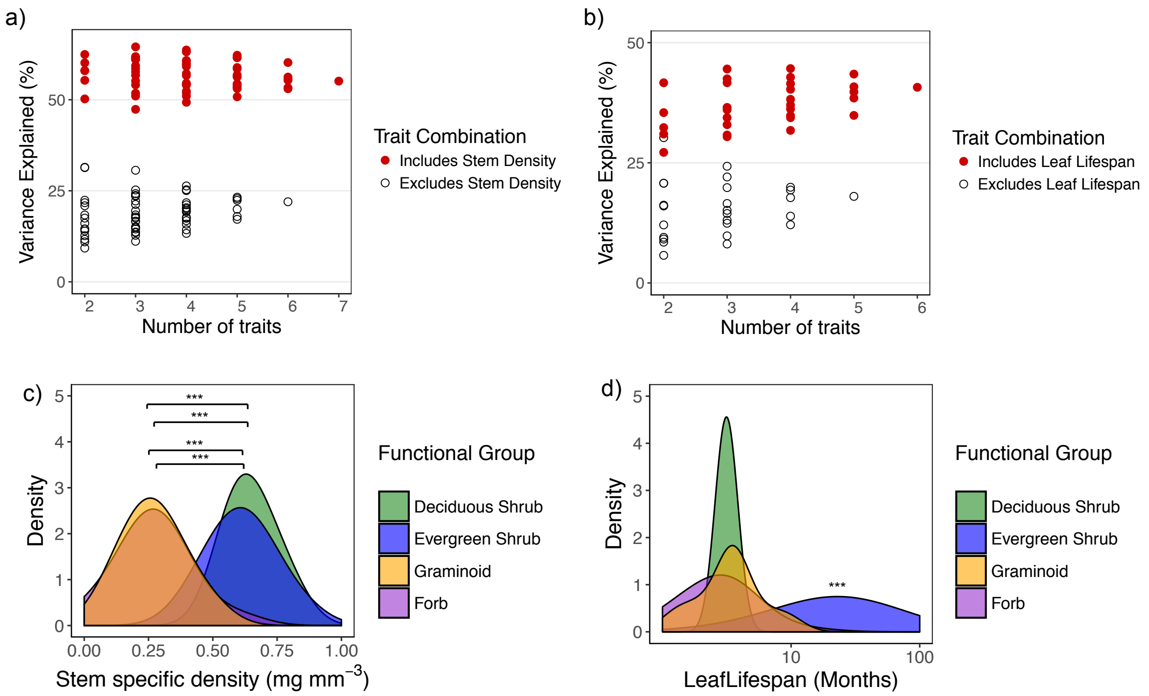

Figure S4 Variation in multivariate trait expression explained by traditional functional groups for all possible trait combinations, including a) stem density and b) leaf lifespan. Previous analyses (Díaz *et al.* 2016) have used stem density rather than leaf dry matter content, but this trait was unavailable for the majority of tundra species and is highly correlated with LDMC (Büntgen, Psomas & Schweingruber 2014). Inclusion of stem density increases explanatory power of functional groups to 55%, but stem density data are available for only 53 species and so may not represent biome-scale patterns. trait data for some species. Inclusion of leaf lifespan increases explanatory power of functional groups to 41%, but leaf lifespan data have only146 available observations across 102 species, and so also may not represent biome-scale patterns. Distribution of tundra plant traits represented by the four traditional tundra plant functional groups for c) stem specific density and d) leaf lifespan reveal that differences among groups are driven by shrub vs non-shrub species (stem specific density), and evergreen shrubs (leaf lifespan). Trait values are presented on the x axis in untransformed units on a log scale.

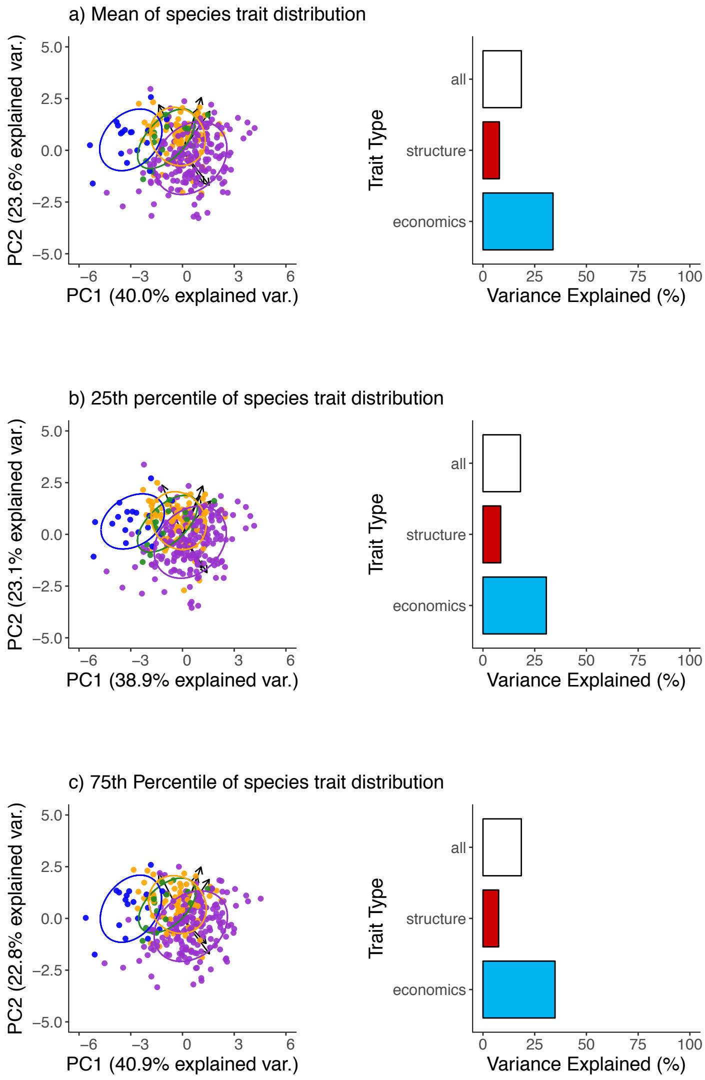

Figure S5 Variance explained by traditional functional groups is consistent across **a**) the species-level mean of trait distributions (main analysis), **b**) the 25th percentile of species-level trait distributions, and **c**) the 75th percentile of species-level trait distributions. Functional space was defined based on plant height (PH), seed mass (SM), leaf area (LA), specific leaf area (SLA), leaf dry matter content (LDMC) and leaf nitrogen content (LN). Individual species are represented by points and functional groups by point colour (blue = evergreen shrub, green = deciduous shrub, yellow = graminoid, purple = forb). Ellipses represent 95% confidence interval of functional group distributions. Arrows indicate direction and weighting of each trait.

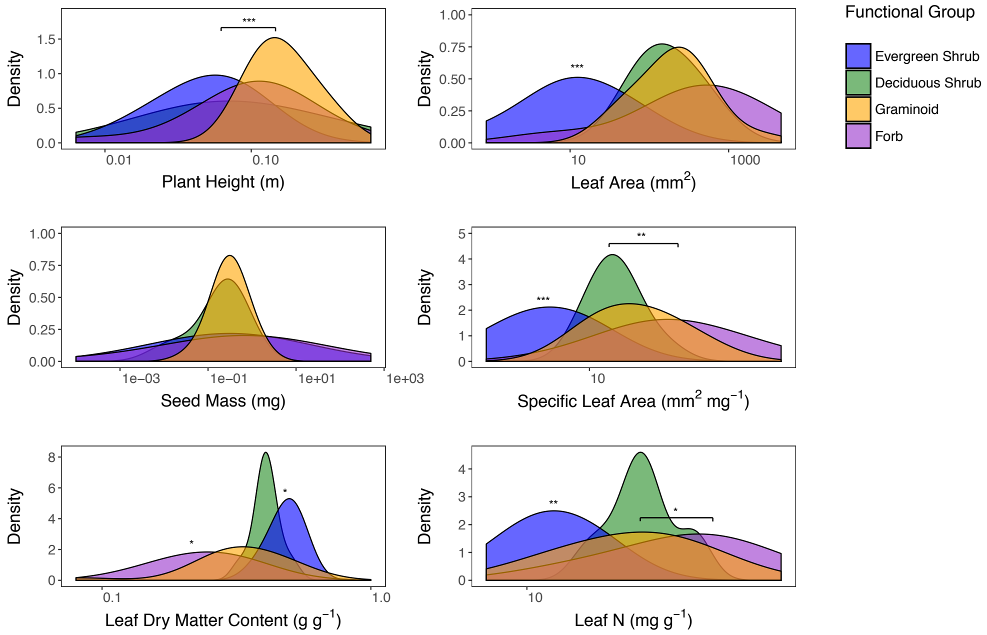

Figure S6 – Distribution of species-level traits represented by the four traditional tundra plant functional groups. Distributions are based on species-level mean traits using only georeferenced trait data from locations north of 60°N. Note that only 57 tundra species have available trait data from these locations (compared to 295 species using the full dataset). Trait values are presented on the x axis in untransformed units on a log scale. Significance of distributions is indicated by symbols (pairwise wilcox test; * = $P < 0.05$; ** = $P < 0.01$, *** = $P < 0.001$).

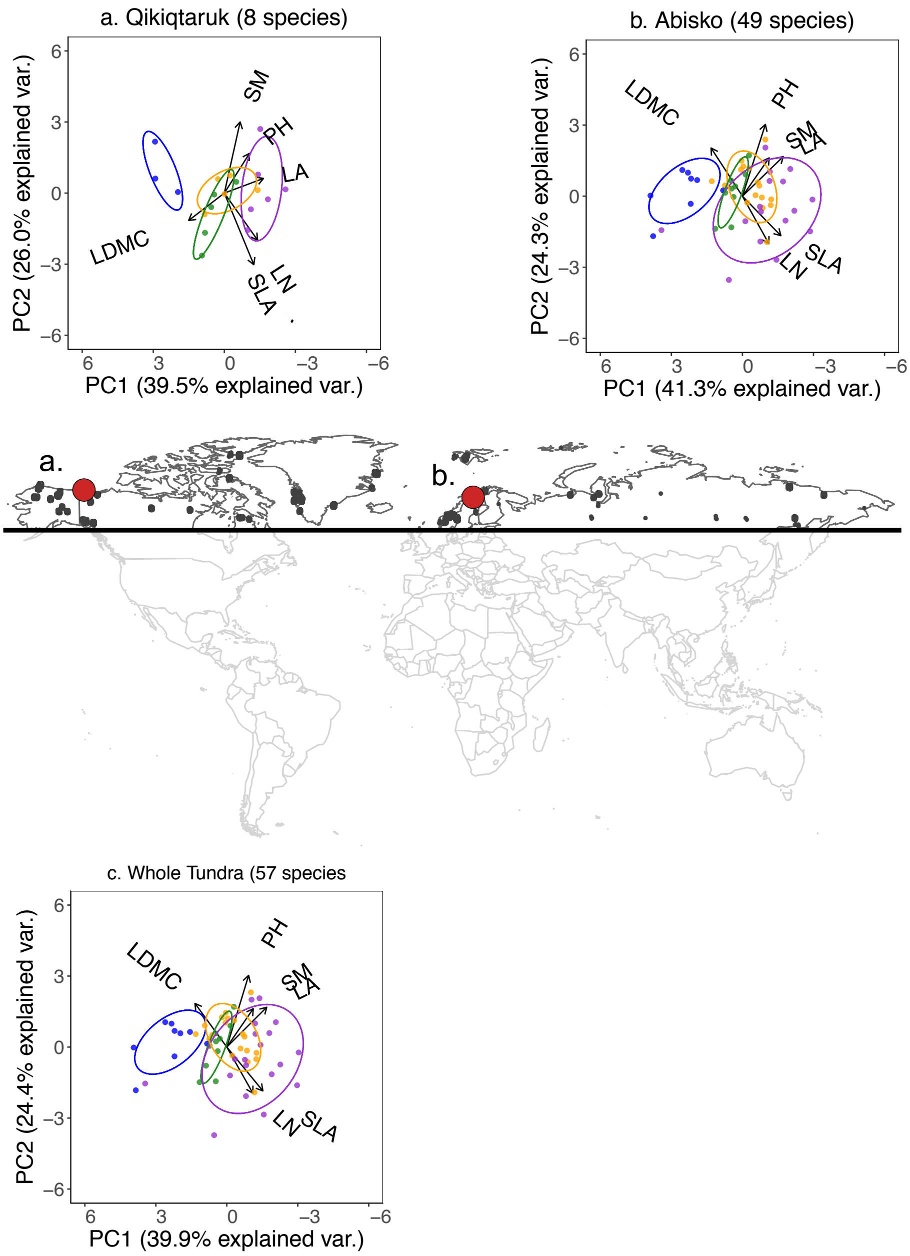

Figure S7 Distribution of tundra species in functional trait space using only georeferenced trait data from locations north of 60°N. Note that only 57 tundra species have available trait data from these locations compared to 295 species using the full dataset. Inset plots indicate PCA multivariate distribution of six plant traits for two tundra sites (**a**) Qikiqtaruk, (**b**) Abisko, and for **c)** the Arctic tundra. Georeferenced trait collection locations are indicated by grey circles and modelled site locations by red circles. Functional space was defined based on plant height (PH),

seed mass (SM), leaf area (LA), specific leaf area (SLA), leaf dry matter content (LDMC) and leaf nitrogen content (LN). Individual species are represented by points and functional groups by point colour (blue = evergreen shrub, green = deciduous shrub, yellow = graminoid, purple = forb). Ellipses represent 95% confidence interval of functional group distributions. Arrows indicate direction and weighting of each trait.

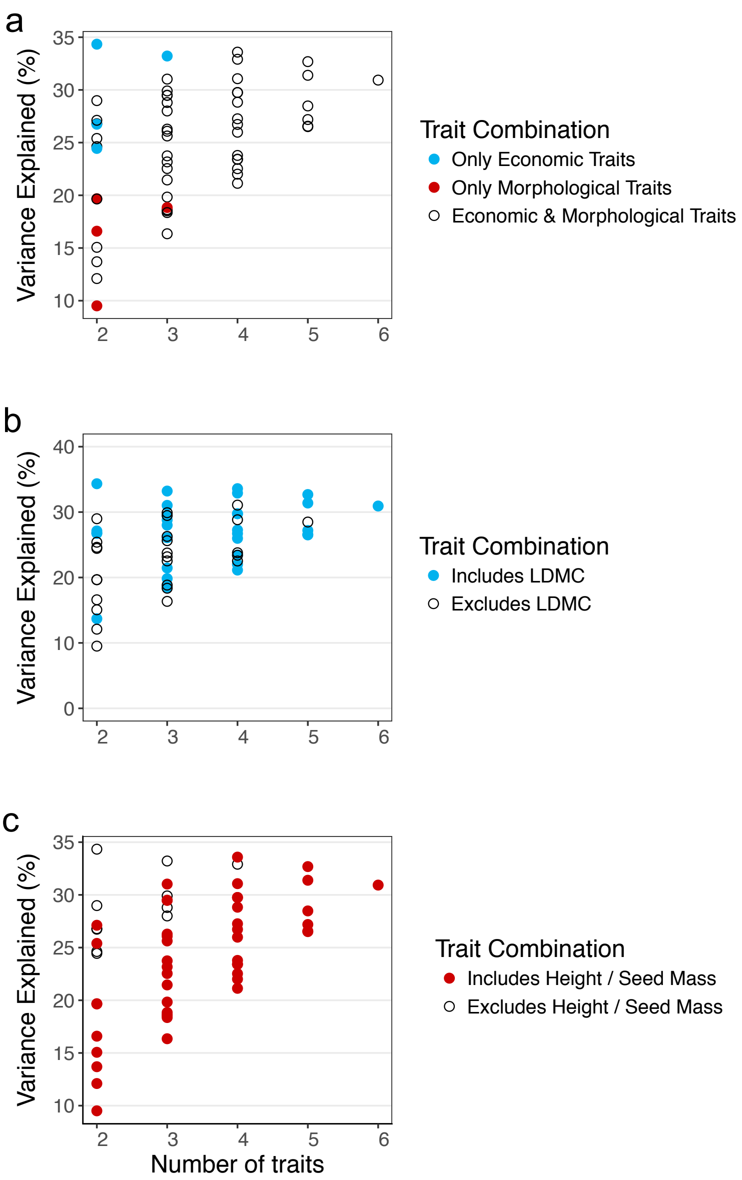

Figure S8 Trait variation explained by traditional functional groups for all possible trait combinations using only georeferenced trait data from locations north of 60°N. Functional groups best explain combinations of only economic traits (**a**) or those containing leaf dry matter content (LDMC) (**b**), and worst explain combinations of only morphological traits (**a**) or those containing plant height or seed mass (**c**). Note that only 57 tundra species have available trait data from these locations (compared to 295 species using the full dataset). Points indicate the mean variance explained

(PERMANOVA R^2) by functional groups and coloured to visualise the importance of different trait combinations.

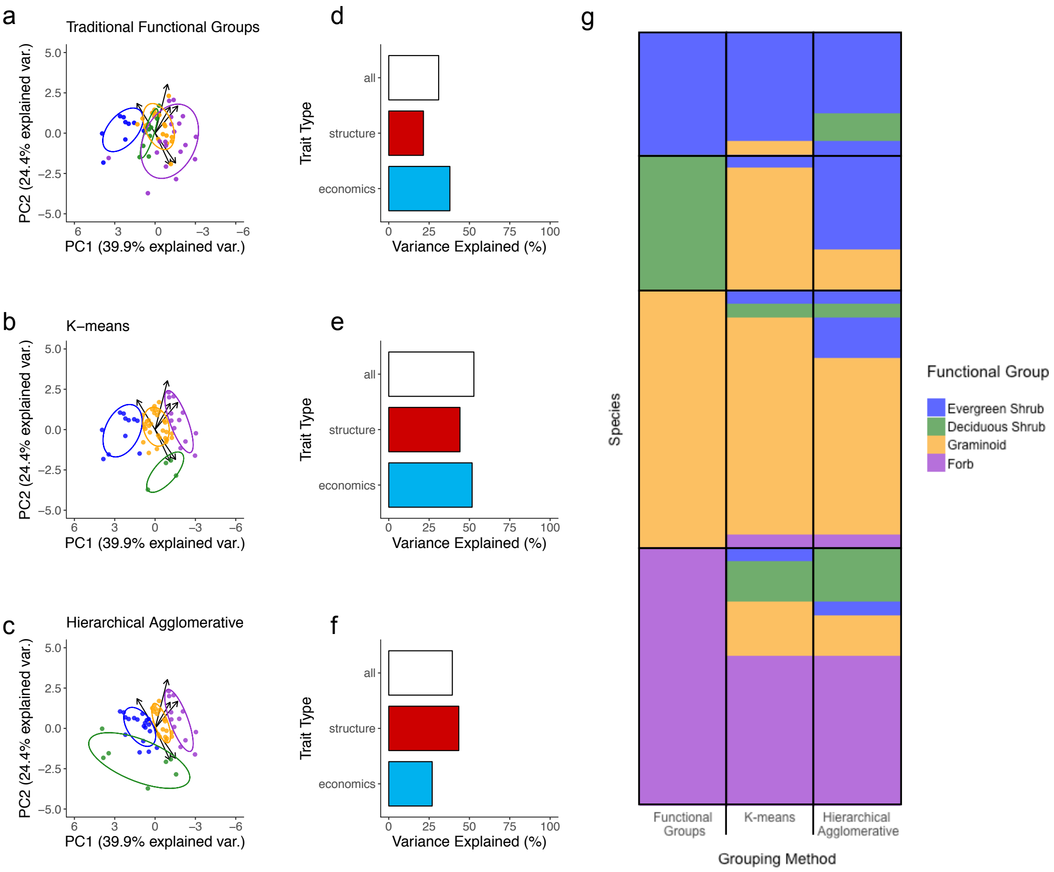

Figure S9 Comparison of group structure, trait variation explained, and group composition between traditional functional groups and *post-hoc* classifications using only georeferenced trait data from locations north of 60°N. Note that only 57 tundra species have available trait data from these locations (compared to 295 species using the full dataset). (**a-c**): PCA visualization of species clusters as defined by (**a**) traditional functional groups, (**b**) k-means clustering, and (**c**) hierarchical-agglomerative clustering (HCA). Species are indicated by points and group distribution by ellipses. Colours indicate groups (dark blue = evergreen shrub, green = deciduous shrub, yellow = graminoid, purple = forb). *Post-hoc* classifications are matched with functional groups based on maximum species correspondence between grouping methods, such that each *post-hoc* classification corresponds with a traditional functional group. (**d-f**): Trait variation explained by (**d**) traditional functional groups, (**e**) k-means, and (**f**) HCA for multivariate combinations of all six

plant traits (white), structural traits only (red), and economic traits only (light blue). (**g**): Comparison of group composition across clustering methods. The stacked bars represent individual species and are ordered by traditional functional group (species order remains consistent across columns). The colour of each stacked bar represents the group to which species were assigned by each classification method (classification can change across columns). For example, a species categorised as a graminoid by traditional functional groups can be categorised in the group most corresponding to forbs by *post-hoc* classifications.

Supplementary references

Büntgen, U., Psomas, A. & Schweingruber, F.H. (2014) Introducing wood anatomical and dendrochronological aspects of herbaceous plants: Applications of the Xylem Database to vegetation science. *Journal of Vegetation Science*, **25**, 967–977.

Díaz, S., Kattge, J., Cornelissen, J.H.C., Wright, I.J., Lavorel, S., Dray, S., Reu, B., Kleyer, M., Wirth, C., Prentice, C.I., Garnier, E., Bönisch, G., Westoby, M., Poorter, H., Reich, P.B., Moles, A.T., Dickie, J., Gillison, A.N., Zanne, A.E., Chave, J., Joseph Wright, S., Sheremet'ev, S.N., Jactel, H., Baraloto, C., Cerabolini, B., Pierce, S., Shipley, B., Kirkup, D., Casanoves, F., Joswig, J.S., Günther, A., Falczuk, V., Rüger, N., Mahecha, M.D. & Gorné, L.D. (2016) The global spectrum of plant form and function. *Nature*, **529**, 167–171.
